# A 20-year journey in transcatheter aortic valve implantation: Evolution to current eminence

**DOI:** 10.3389/fcvm.2022.971762

**Published:** 2022-11-21

**Authors:** Andreas S. Kalogeropoulos, Simon R. Redwood, Christopher J. Allen, Harriet Hurrell, Omar Chehab, Ronak Rajani, Bernard Prendergast, Tiffany Patterson

**Affiliations:** ^1^St. Thomas’ Hospital, Guy’s and St. Thomas’ NHS Foundation Trust, London, United Kingdom; ^2^Department of Cardiology, MITERA General Hospital, Hygeia Healthcare Group, Athens, Greece; ^3^School of Bioengineering and Imaging Sciences, King’s College London, London, United Kingdom

**Keywords:** TAVI, TAVR, aortic stenosis (AS), paravalvular aortic leak, bicuspid and tricuspid aortic valve, aortic valve calcification, minimalistic approach

## Abstract

Since the first groundbreaking procedure in 2002, transcatheter aortic valve implantation (TAVI) has revolutionized the management of aortic stenosis (AS). Through striking developments in pertinent equipment and techniques, TAVI has now become the leading therapeutic strategy for aortic valve replacement in patients with severe symptomatic AS. The procedure streamlining from routine use of conscious sedation to a single arterial access approach, the newly adapted implantation techniques, and the introduction of novel technologies such as intravascular lithotripsy and the refinement of valve-bioprosthesis devices along with the accumulating experience have resulted in a dramatic reduction of complications and have improved associated outcomes that are now considered comparable or even superior to surgical aortic valve replacement (SAVR). These advances have opened the road to the use of TAVI in younger and lower-risk patients and up-to-date data from landmark studies have now established the outstanding efficacy and safety of TAVI in patients with low-surgical risk impelling the most recent ESC guidelines to propose TAVI, as the main therapeutic strategy for patients with AS aged 75 years or older. In this article, we aim to summarize the most recent advances and the current clinical aspects involving the use of TAVI, and we also attempt to highlight impending concerns that need to be further addressed.

## Introduction

Since the first groundbreaking procedure in 2002, transcatheter aortic valve implantation (TAVI) has led to a pervasive transformation in the management of severe aortic stenosis (AS). TAVI has now been shown to be non-inferior or even superior to surgical aortic valve replacement (SAVR) in several important randomized clinical trials (RCTs) across the whole spectrum of surgical risks, including high-, intermediate-, and low-risk patients. The procedure streamlining with the introduction of new generation transcatheter heart valve (THV) design, the establishment of dedicated computed tomography (CT) TAVI analysis for pre-procedural planning (valve and arterial access selection), the better patient selection, the minimalization of the procedure (single arterial access and conscious sedation), the transition from dual to single antiplatelet therapy and several technical enhancements (cusp overlap technique for self-expanding THVs) have driven a dramatic improvement on outcomes and safety of the procedure and also an even more vivid reduction of procedural complications over time. These advances have opened the road to the use of TAVI in younger and lower-risk patients, leading to an expansion of current guideline recommendations for TAVI. As TAVI rapidly expands to younger and lower-risk patients with longer life expectancy, new concerns of paramount significance have emerged, such as THV durability in comparison with surgical bioprostheses, coronary access after TAVI, paravalvular regurgitation, the prognostic impact of conduction disturbances, and need for re-intervention after TAVI. In this review, we aim to summarize the most recent advances and the current clinical aspects involving the use of TAVI and we also attempt to highlight impending concerns that need to be further addressed.

### Evolution and contemporary perceptions on transcatheter aortic valve implantation: Vascular access, the minimalist approach, and the fast-track discharge pathways

**FIGURE 1:**
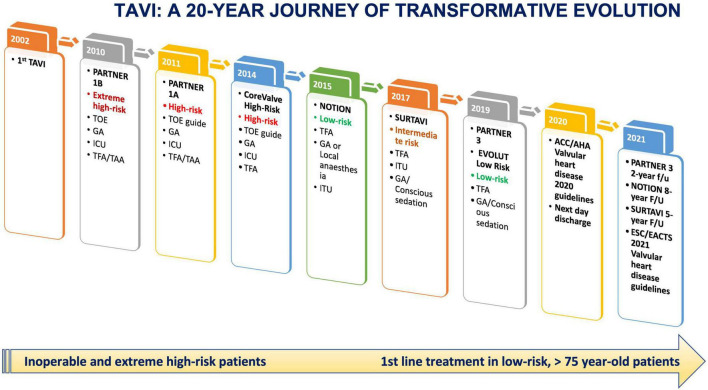
TAVI: A 20-year journey of transformative evolution from high-risk inoperable patients to the most recent European and US guidelines and low-risk younger patients along with landmark trials.

**FIGURE 2:**
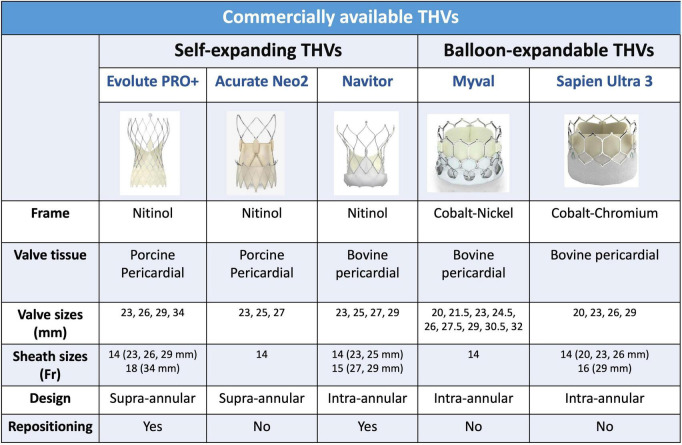
Commercially available transcatheter aortic valves.

After two decades of clinical experience, the TAVI procedure has undergone a transformative evolution (Figure 1). The new generation THVs with improved sizing, deliverability, and positioning compared to their predecessors (Figure 2), the advent of new hydrophilic, small bore, expandable and atraumatic sheaths, as well as the introduction of intravascular lithotripsy have now made transfemoral TAVI feasible in > 95% of patients.

Pre-procedural planning including valve selection and vascular access has been refined by standardization of CT imaging, which has now been established as the ultimate imaging modality for evaluating vascular access, annular dimensions, and valve morphology, and predicting potential complications, such as acute coronary occlusion, annular rupture, and conduction disturbances (1). In addition, the introduction of newly developed and sophisticated 3D software simulating procedural outcomes such as the severity of the paravalvular leak and the need for pacemaker (PPM) implantation will help to further improve TAVI procedural outcomes (FEops HEART Guide™, Gent, Belgium) (2).

**FIGURE 3:**
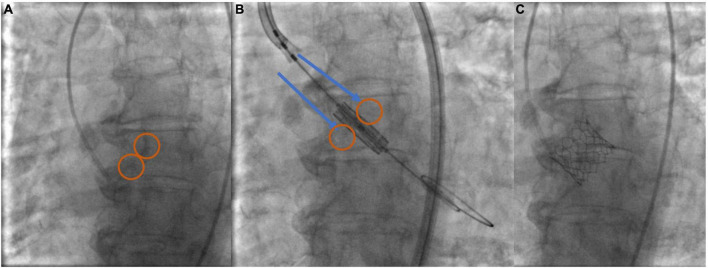
Minimalist—single arterial access technique implantation using aortic valve leaflet calcification for THV positioning and deployment. **(A)** Identify calcium markers and annular plane in 3-cusp view (circled). **(B)** Position Sapien ULTRA 3 central balloon marker—align with annular plane and calcium marker (arrows-circles). **(C)** THV deployment.

A growing proportion of TAVI cases worldwide are now performed using a “minimalist” approach, which incorporates conscious sedation (CS), local anesthesia, and a post-procedure transthoracic echocardiographic assessment. Conscious sedation is commonly used across Europe and has conceivable advantages including reduced procedural time, faster recovery, and reduced cost and it is also associated with a shorter hospital stay and reduced short-term mortality (3, 4). Moreover, the transition from secondary femoral to radial access for guiding valve deployment and assessing the vascular closure of the primary access has further simplified TAVI and has substantially reduced the risk of vascular complications (5). A newly introduced minimalistic technique incorporating a single arterial transfemoral access and the use of aortic valve leaflet calcifications as the fluoroscopic markers for THV positioning has shown promising results as a safe and effective approach associated with a lower rate of complications, procedural time, and contrast volume during the implantation of the Sapien 3 THV system (Figure 3) (6).

Over the last decade, different tools such as the micro-puncture kit, the ultrasound (US) guided vascular access, and the newly introduced intravascular lithotripsy has been associated with reduced peri-procedural vascular complications and has expanded the feasibility of transfemoral TAVI in patients with peripheral vasculopathy. Precise selection of the femoral cannulation site, pointing to avoid sites of anterior calcification, and successful implantation of percutaneous closure devices is of paramount importance in reducing vascular complications. US guidance allows a real-time examination of the vessel wall and the selection of the ultimate puncture area by identifying conventional landmarks, such as the femoral bifurcation (below) between the superficial femoral artery and the profunda femoris and the inguinal ligament (upper). The ultimate area of cannulation is in the horizontal segment of the common femoral artery (CFA), in the middle of the anterior wall in an area free of calcium. This technique has consistently been shown to improve puncture success rates at the first attempt, reduce accidental venipuncture rates, increase physician confidence, and reduce patients’ life-threatening bleeding complications (7). In addition, the use of dedicated micro-puncture 21-gauge (G) needles with a US visible tip has been shown to reduce the rate of vascular complications with a significant decrease in the number of groin hematomas compared to standard large bore needles (8, 9).

Almost 35% of the elderly population undergoing TAVI procedures suffer from peripheral vascular disease with tortuous and heavily calcified vessels (10). Non-calcified arteries can be stretched, and successful insertion of TAVI delivery arterial sheaths can be achieved with an arterial lumen as small as 75% of the TAVI sheath’s outer diameter. In contrast, for calcified and tortuous vessels, it is highly recommended that the minimum lumen diameter should be at least 1.25 mm bigger than the sheath. For the 14 or 16 F inner diameter sheaths of the contemporary miniaturized delivery systems, this is equal to minimum diameters of approximately 6–7 mm in non-calcified and calcified vessels, respectively (11). In this context, the newly introduced Shockwave intravascular lithotripsy balloon catheter (IVL) (Shockwave Medical Inc., Santa Clara, CA, USA) has emerged as a promising tool for lesion preparation as an elective or bailout strategy in patients with severe peripheral vascular disease intended for TAVI but considered ineligible for transfemoral access (12–14).

This transformational evolution of TAVI has led to a dramatic reduction in procedural mortality and major complication rates. Data from the UK TAVI registry have shown a dramatic reduction in in-hospital mortality after TAVI (9.09% in 2009 to 1.84% in 2016) (15). In addition, similar reductions in mortality and complication rates have been observed in large data series from other registries in France, Germany, Japan, and the USA (16–19). More particularly, the incidence of stroke dropped from 3.4 to 2.2%, acute kidney injury requiring dialysis from 6.4 to 0.9%, and cardiac tamponade from 5.3 to 1.4%. These improved outcomes were also associated with reduced in-hospital stay, with the median time from procedure to discharge falling from 130 h (2013) to 64 h (2016) (20). An all-corners patients’ retrospective analysis has shown that a fast-track median length of post-TAVI in-hospital stay of 3-days compared to a standard 6-day in-hospital stay did not have any difference in all-cause mortality (1.3 vs. 1.9%), rate of rehospitalization after discharge (2.09 per patient-year vs. 2.09 per patient-year) and rate of permanent pacemaker implantation (PPI) in pacemaker naive patients at 90 days (15.8 vs. 21.9%) (21). In addition, two prospective studies the 3M-TAVR and FAST-TAVI have shown that next-day discharge is safe in judiciously selected patients who undergo uncomplicated transfemoral TAVI (22, 23). This is likely to further fall with dedicated and vigilantly structured early-discharge pathways as it has been brilliantly illustrated during the COVID-19 pandemic and the subsequent bed pressure to hospitals to further push their boundaries. Two recent studies have shown that in a selected population of TAVI patients with either *in situ* PPM or low risk for conduction abnormalities same-day discharge was feasible and safe (24, 25).

## Patients’ selection—The choice between transcatheter aortic valve implantation and surgical aortic valve replacement

Over the last decade, TAVI has led to a paradigm shift in the treatment of symptomatic severe AS and has now established itself as the treatment of choice in patients with symptomatic severe AS across all risk categories. The publication of the randomized trials PARTNER 3 (26) and the Evolut Low-Risk study (27) confirmed favorable outcomes of TAVI compared to SAVR even in patients with symptomatic AS at low surgical risk [Society of Thoracic Surgeons (STS) risk score < 4%]. The PARTNER 3 (Placement of Aortic Transcatheter Valve) trial highlighted the superiority of Transfemoral TAVR with the third-generation balloon-expandable SAPIEN 3 valve (Edwards Lifesciences LLC, Irvine California) over SAVR in 1,000 patients with mean STS risk score of 1.9%, for the primary endpoint of death from any cause, stroke, or rehospitalization (26). These results were also confirmed at a 2-year follow-up [TAVI: 11.5% vs. SAVR: 17.4%; Hazard Ratio (HR): 0.63; 95% CI: 0.45–0.88; *P* = 0.007]. TAVI was also associated with a lower incidence of disabling stroke at 30 days and new-onset atrial fibrillation (AF), with no significant differences between groups in major vascular complications, new PPM implantation, and moderate or severe paravalvular regurgitation (28). However, this trial did not include patients with bicuspid aortic valve (BAV) or other complex high-risk aortic valve anatomies, significant coronary artery disease, low-flow low-gradient AS, concomitant valve disease, peripheral vascular disease precluding transfemoral access, and therefore, its findings cannot be extended or applied at these cohorts. In addition, at 2 years, the TAVI group demonstrated a signal for the significantly higher incidence of subclinical valve thrombosis (2.6 vs. 0.7%) and numerically higher mean gradients and lower effective orifice area. Whether these findings will reflect a more likely route of earlier valve failure, we will need to wait for more to see the results of the long-term follow-up outcomes of the study at 10 years.

The Evolut Low Risk study randomized 1,468 patients at low surgical risk to either TAVI with self-expanding supra-annular CoreValve, Evolute R, or Evolut PRO (Medtronic Inc. Minneapolis) or SAVR. At 2 years, the study showed non-inferiority of TAVI vs. SAVR for the primary composite endpoint of all-cause death or disabling stroke (TAVI: 4.3% vs. SAVR: 6.3%, *P* = 0.084 for superiority; *P* < 0.001 for non-inferiority). At 30 days, TAVI was associated with lower rates of disabling strokes, bleeding complications, acute kidney injury, and AF. As far as the THV performance is concerned, supraannular Evolut THV was associated with lower transvalvular gradients, larger effective valve area, and less frequent prosthesis-patient mismatch than SAVR, but more frequent mild and moderate PVL (27). In addition, at 8-years follow-up, in a low-risk population, the NOTION trial has shown comparable mortality between the Evolut self-expandable platform and surgery (51.8 vs. 52.6%, *p* = 0.94). Moreover, the rate of structural valve deterioration (SVD) was substantially lower with TAVI (13.9 vs. 28.3%; *p* = 0.017) (29). However, several issues should be considered before we attempt to extrapolate these results to the more general population with AS. The overall number of patients that were still alive at 8 years follow-up was very small, 133 patients from which 12 did not reach the 8-year follow-up visit. As the trial was initially designed to evaluate the primary outcome at 1-year follow-up, the results of the 8-year follow-up comprise an exploratory only and not conclusive analysis. In addition, in the SAVR group, 34% of the patients received Mitroflow and Trifecta bioprostheses, which have been consistently reported to have a higher risk of earlier SVD. Although the risk of SVD was significantly lower after TAVI, when compared with SAVR, the definition of SVD included several imaging findings that do not necessarily result in clinical symptoms or do impose further intervention. Besides, the rate of the more clinically important bioprosthetic valve failure (BVF) was comparable between TAVI and SAVR groups.

**FIGURE 4:**
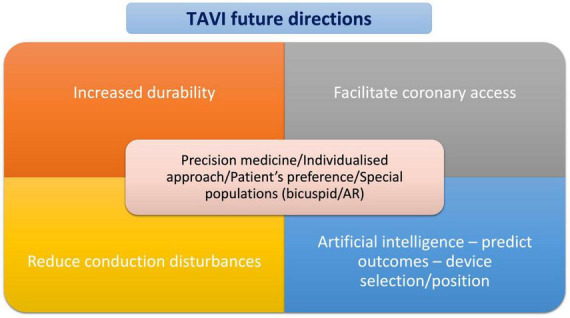
TAVI future directions.

Further corroborating the results of the above RCTs that have demonstrated comparable or even superior clinical outcomes between TAVI and SAVR in the low-risk population with severe AS, a recent pooled meta-analysis of aggregated data of 8,020 patients showed that within a follow-up period of 2 years, TAVI was associated with a significant reduction of all-cause mortality compared to SAVR [Hazard Ratio (HR): 0.88, 95% CI: 0.78–0.99, *P* = 0.030; an effect that was consistent across the entire spectrum of surgical risk (*P*-for-interaction = 0.410) and irrespective of the type of the THV system (*P*-for-interaction = 0.674)]. The TAVI was also associated with a lower risk for stroke [Hazard Ratio (HR): 0.81, 95% CI: 0.68–0.98, *P* = 0.001] (30). In line with the new data, the 2021 ESC guidelines on the management of severe AS recommended transfemoral TAVI as the first-line therapy in patients older than 75 years old or those at high risk (STS PROM/EuroSCORE II > 8%) or unsuitable for surgery and it is recommended for remaining patients according to individual clinical, anatomic, and procedural characteristics (Class I) (31). The 2020 ACC/AHA guidelines recommend TAVI in preference to SAVR for patients with severe symptomatic AS aged > 80 years and in younger patients with a life expectancy < 10 years and no anatomic contraindication to transfemoral TAVI and have endorsed TAVI as Class I for patients with symptomatic severe AS aged 65–80 years from prohibitive to low-surgical risk patients (32). Considering the expanded indications of TAVI to lower-risk and younger patients, a shared decision-making process is strongly advised. The choice between TAVI and SAVR should be made after careful consideration on the patient’s life expectancy and valve durability and should be based upon a meticulous evaluation of the patient’s personal preference, and anatomical and procedural factors, weighing the risks and benefits of each approach for the individual patient (Figure 4).

## Current transcatheter valve devices: Lifetime management and durability

The currently approved available THVs include the balloon-expandable and the self-expandable platforms. The decision-making process regarding the choice of a specific device over another one has become even more challenging after the commercialization of multiple platforms. Most recent platforms for both balloon-expandable (SAPIEN 3 ULTRA) and self-expanding (EVOLUT PRO and EVOLUT PRO +) have external sealing skirts that can effectively reduce paravalvular leak (PVL). Even though Edwards SAPIEN and Medtronic EVOLUT have been the most utilized systems, newer THVs have emerged as valuable alternatives. Figure 2 provides a comparative synopsis of the currently commercially available transcatheter systems. Currently, scarce data are available regarding the direct head-to-head comparisons between different devices. In the CHOICE trial, SAPIEN XT and CoreValve THVs showed similar mortality rates but a higher incidence of more than mild PVL with CoreValve (33). In the PORTICO-IDE trial, the intra-annular Portico valve was found to have comparable rates of death or disabling stroke at 2 years compared to the Edwards SAPIEN and Medtronic EVOLUTE systems, but it was associated with higher rates of the primary composite safety endpoint including death at 30 days (34). A head-to-head comparison between the balloon-expandable SAPIEN 3 and the self-expanding EVOLUT R valves was performed in the recently published SOLVE-TAVI trial. Both valves were found to have statistically equivalent performance regarding all-cause mortality (2.3 vs. 3.2%). However, SAPIEN 3 was associated with numerically lower rates of PPM implantation (19.2 vs. 23.0%) and moderate to severe paravalvular leak (1.5 vs. 3.4%) but numerically higher rates of stroke (4.7 vs. 0.5%) (35). TAVI with the self-expanding ACURATE-neo did not meet non-inferiority compared to the balloon-expandable SAPIEN 3 (SCOPE I) and self-expanding Evolut (SCOPE II) in terms of early safety and clinical efficacy outcomes at 1 year (36, 37).

As the patients that were included in the early landmark TAVI trials were mostly elderly with short life expectancy, the THV performance was only evaluated at the short- and mid-term range of follow-up. As the TAVI has now been approved for the treatment of younger and low-risk patients, data collection regarding THV durability has become of utmost importance. SVD is defined as intrinsic permanent changes to the prosthetic valve, including wear and tear, leaflet disruption, flail leaflet, leaflet fibrosis and/or calcification, or strut fracture or deformation (38). The durability of valve bioprosthesis is determined by various physical aspects, such as THV tissue characteristics, anticalcification treatments, leaflet, and valve design and transvalvular gradients, and clinical factors such as patients’ age and various metabolic abnormalities (end-stage kidney disease). In addition, the fundamental difference between SAVR, where the calcified valve is excised completely, and TAVI, where the THV frame is pressed into the calcified valve, may additionally lead to significant differences in fluid dynamics within the sinus of Valsalva affecting long-term bioprosthesis durability. The THVs are also exposed to crimping stress and to a different pattern of stent and leaflet stress.

In an echocardiographic follow-up of patients in the PARTNER 2A trial treated with the SAPIEN XT valve and in the SAPIEN-3 registry, there was inferior durability of the SAPIEN XT vs. the surgical valve with a 2.5-fold rate of SVD in the mid-term follow. Compared with SAVR, the SAPIEN XT TAVI cohort exhibited significantly higher 5-year incidence rates of SVD, SVD-related BVF, and all-cause (structural or non-structural) BVF. The results of the PARTNER 2A trial showed a higher rate of re-intervention within 5 years after the index procedure for the SAPIEN XT, 3.2 vs. 0.6%. In the SAPIEN-3 registry; however, the SAPIEN 3 bioprosthesis had similar rates of SVD (3.9 vs. 3.5%) and SVD-related BVF 1.1 vs. 0.8%) compared to SAVR at 5-year follow-up (39).

Data from the UK TAVI registry showed excellent THV performance and a low incidence of SVD 5–10 years after TAVI. Moderate SVD was noticed in 8.7% of the study population (regurgitation in 57% and stenosis in 43%), whereas severe SVD was noticed in only 0.4% of the study population (40). The investigators of the NOTION study reported sustained clinical outcomes at 8 years after TAVI with self-expandable CoreValve. All-cause death at 8-year follow-up was similar in both groups (TAVR 54.5% vs. SAVR 54.8%). In addition, moderate or severe SVD was significantly higher after surgery (28.3 vs. 13.9%) (29).

**FIGURE 5:**
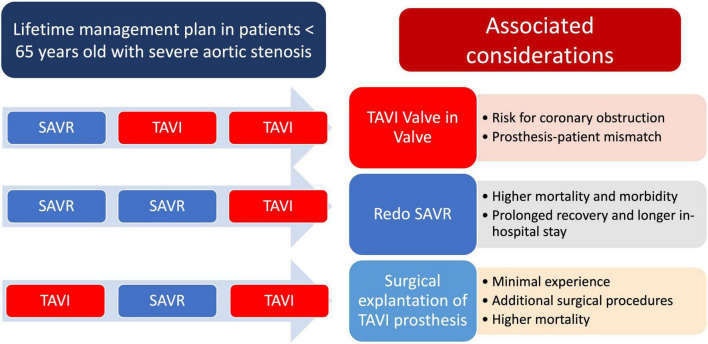
Lifetime management for younger patients < 65 years old with severe aortic stenosis. Potential interventional scenarios and associated considerations. SAVR, surgical aortic valve replacement; TAVI, transcatheter aortic valve implantation.

Given the absence of robust data regarding the long-term durability of either surgical or transcatheter BHVs, it is mandatory that in younger patients with life expectancy > 15–20 years, a careful life management plan should be incorporated as the likelihood of these patients undergoing two or more interventions is high (Figure 5). It is desirable that the number of surgical open-heart interventions should be minimized considering the tending preference of most patients for less invasive procedures and the higher operative mortality and morbidity of redo SAVR compared to SAVR in a native valve (41). In this line, incorporating TAVI in the sequence of long-term interventions makes this strategy more realistic and attractive. Redo SAVR and valve-in-valve (ViV) TAVI are both feasible options. If redoing SAVR is expected in patients in their 60s, a potential SAVR-SAVR-TAVI strategy with the TAVI taking place in 70s–80s is a reasonable approach. On the other hand, a less invasive approach with a single open-heart surgery as the initial strategy followed by ViV TAVI (SAVR-TAVI-TAVI) or TAVI-SAVR-TAVI as an alternative single surgery sequence are potential alternative scenarios with the need for only one open-heart surgery during a lifetime, which makes these options intuitively more attractive to the patients. However, in both these strategies, several issues should be considered and discussed with the patient before implementing a lifetime management plan. In the case of SAVR after TAVI, depending on the type of BHV implant at the index TAVI, surgical explantation of the valve may require additional procedural steps and more extensive surgery, such as root replacement and/or replacement of the ascending aorta. The largest so far observational analysis with 5,756 patients with previous TAVI, the incidence of redo SAVR after TAVI was 0.5% with the most frequent indication being infectious endocarditis (67.8% of patients). For most patients, 60.7% required additional cardiac surgical procedures and the overall 12-month mortality was 33.5% (42). On the other hand, ViV TAVI is associated with several considerations, including the risk of coronary obstruction, prosthesis-patient mismatch, and the need for previous surgical bioprosthesis cracking. However, according to a recent large-scale meta-analysis and observational study, ViV TAVI was associated with lower 1-month mortality, a noteworthy threefold reduction in bleeding and respiratory complications, and less in-hospital stay with faster recovery compared to redo SAVR (43, 44). Novel technologies that will further improve the durability of BHVs will facilitate lifetime management plans for younger patients with severe AS. As such the recently presented RESILIA bioprosthetic leaflet tissue (Edwards Lifesciences), which has already been applied in surgical bioprostheses (INSPIRIS RESILIA aortic valve bioprosthesis, Edwards Lifesciences) and now has also been introduced in the new generation Sapien TAVI bioprosthesis has demonstrated excellent 5-year outcomes with no evidence of SVD after SAVR (45).

## Transcatheter aortic valve implantation in particular patients’ cohorts

### Transcatheter aortic valve implantation in degenerated surgical bioprostheses (valve-in-valve transcatheter aortic valve implantation)

Mainly due to the aging population and increased use of bioprosthetic rather than mechanical valves, the need for redo intervention in the context of degenerative bioprosthetic valve disease has substantially increased over the last few years. ViV TAVI has emerged as an appealing alternative to the surgical approach for the treatment of failed surgical and transcatheter bioprosthetic valves, mainly due to the higher risk of periprocedural complications of redo SAVR compared to *de novo* surgery. The patients’ frailty and the high burden of underlying comorbidities make ViV-TAVI a reasonable, less invasive, and much more attractive option for patients with degenerated bioprostheses. In the last few years, an incremental trend in both strategies has been observed with significantly more frequent utilization of TAVI ViV rather than redoing SAVR (46). Balloon expandable THVs showed higher rates of patient-prosthesis mismatch compared to self-expanding platforms in a registry of 459 patients undergoing ViV TAVI (46, 47). Therefore, self-expanding THVs should be considered a preferable option in patients with a small surgical bioprosthesis. Even though the difference was not statistically significant, a trend for lower mortality has been observed with ViV-TAVI compared to redo SAVR. Furthermore, ViV-TAVR has been associated with fewer in-hospital major adverse cardiovascular events (MACE) and reduced hospitalizations (46). These findings have been further confirmed in a large-scale metanalysis, including 23 studies and 8,509 patients. Compared to redo SAVR, ViV-TAVI was associated with no significant differences in 30-day mortality and stroke rates and 1-year mortality, suggesting a potential superiority of ViV-TAVI as the 1st line treatment for degenerative BVF. In the more recent 5-year follow-up of PARTNER II—Nested Registry/Valve in Valve study, TAVI for bioprosthetic aortic valve failure was associated with improved survival, valve hemodynamics, and, more importantly, sustained quality-of-life outcomes (48). Updated follow-up of the VIVID Registry reported the longest follow-up on a large scale of patients at high surgical risk with an estimated survival at 5 years of 38% (49). In a direct comparison of re-SAVR patients, ViV-TAVI patients had significantly lower 30-day mortality (2.7 vs. 5.0%), 30-day morbidity (66.4 vs. 79%), and rates of major bleeding (35.8 vs. 50%) (50). ViV TAVI was also associated with a shorter length of stay and higher odds of routine home discharges compared to re-SAVR (50). Another important issue in ViV TAVI is the risk for acute coronary obstruction, a life-threatening complication that can occur in 2.3% of patients undergoing TAVI ViV. The primary mechanism behind acute ostial coronary occlusion after ViV TAVI is a leaflet of the prior valve displacement toward coronary ostia, resulting in an obstruction of coronary blood flow. Even though multiple reports have demonstrated the feasibility of intentional bioprosthesis leaflet laceration with electrocautery wire (BASILICA) as a potential technique to prevent acute coronary occlusion after ViV TAVI, this technique is challenging, not widely adopted, and can be associated with potential risks. A novel dedicated device, the ShortCut (Pi-Cardia) device, is the first dedicated device specifically designed for the precise and controlled laceration of the bioprosthetic aortic valve leaflet imposing the risk for acute ostial coronary occlusion after ViV TAVI (51).

Optimal pre-procedural planning and then procedural execution, through a methodological and a step-by-step approach, have a fundamental role in achieving an optimal result after a ViV TAVI. Successful ViV TAVI requires correct identification of the previous surgical valve, the selection of an appropriate THV, and the implantation of the latter in the correct position (52). A ViV application tool (ViV Aortic) by Bapat (53) is available at online app stores and has been specifically developed to aid the interventionalist in choosing the transcatheter device suitable for the various surgical bioprostheses.

### Transcatheter aortic valve implantation in bicuspid aortic valve

BAV represents the most common congenital cardiac anomaly with an estimated incidence of 2% accounting for approximately 50% of cases requiring SAVR in younger patients (54, 55). BAV is known to exhibit a very heterogeneous morphology with considerable variations in leaflet geometry, leaflet orientation, presence, or absence of raphe, and especially in the severity of calcification of the aortic valve and the adjacent structures. Several schemes have been proposed so far to classify BAV—all of them addressing all these morphological aspects (56–59). Due to the presence of various morphological conditions, there are currently only limited data on which BAV anatomy favors a TAVI procedure, the implantation strategy, and device that will provide optimal results, the sizing strategy that should be applied, and the long-term THV durability in these very heterogeneous settings. However, it is unanimously accepted that severe and asymmetric leaflet and LVOT calcification, the presence of more elliptical aortic annulus that exceeds available sized THVs, a dilated ascending aorta > 45 mm, and the presence of raphe calcification can result in suboptimal THV frame expansion and potentially worsen outcomes.

A contemporary and optimal TAVI technology in a BAV morphology can mitigate the risk of PVL, annular rupture, and the need for second valve implantation (60). Historically, early-generation TAVI devices have performed worse in BAV anatomy, showing worse in-hospital outcomes, decreased device success, and increased incidence of device malpositioning, PVL, and aortic root injury (61). The recent refining of the device iteration has increased TAVI procedural success rates with noticeably improved short- and mid-term outcomes. Data from the STS/ACC TVT registry did not show any difference in 30-day (2.6 vs. 1.7%; *p* = 0.18) or 1-year mortality (10.4 vs. 12.1%; *p* = 0.63) between patients’ propensity-matched cohorts of intermediate surgical risk with bicuspid vs. tricuspid AS undergoing TAVI with a self-expanding TAVI bioprosthesis. Valve hemodynamics appeared outstanding for both bicuspid and tricuspid patients up to 1-year, although post-procedure moderate or severe PVL was more frequent in BAV (5.6 vs. 2.1%; *p* < 0.001) (62). The presence of calcified raphe and excess leaflet calcification have been reported as robust predictors of increased intraprocedural risk and mid-term mortality, highlighting the need for further refinement in device technology and technical aspects to make TAVI a safer procedure for the treatment of BAV stenosis (59). Data from the BEAT registry has shown that new-generation balloon-expandable and self-expandable platforms had comparable clinical outcomes up to 1-year and similar device success. However, the balloon-expandable THV was associated with less PVL (0.8 vs. 10.8%; *p* < 0.001) and higher mean gradients 11.3 mmHg vs. 9.6 mmHg; *p* < 0.001) (63).

Currently, there is no standardized system for the sizing of THV in the setting of a TAVI BAV. In the BAVARD registry, THV sizing was based either on the size of the aortic annular plane or the intercommissural distance of the slit-shaped orifice 4 mm above the annular plane to appropriately select the device and predict the sealing. Annular sizing was recommended in 88% of patients with a tube- or flare-shaped BAV and sizing according to the intercommissural distance in a volcano-shaped BAV (64). On the contrary, other groups showed that supra-annular sizing was less reproducible and did not find any difference in complication rates in patients in whom supra-annular sizing would have altered the device size used (65). Furthermore, an alternative modifying sizing algorithm incorporating the length and calcium load of the raphe combined with the overall volume of calcium in BAV morphology with a raphe has been proposed for TAVI in BAV (66). Based on advanced CT scan analysis, a promising concept of simulating the post-TAVI result, including information on frame deformation, paravalvular regurgitation, and major conduction abnormalities, in a small cohort of BAV patients has been applied with promising results. The investigators were able to accurately identify those patients with a hostile device landing zone for the THVs (67, 68). Even though undersized strategies seem to be more appropriate in some of the BAV patients, rapid, efficient, and reproducible algorithms for the optimal THV device selection do constitute an unmet clinical need and still need to be proven.

### Transcatheter aortic valve implantation in patients unsuitable for transfemoral access

With the evolution of the pertinent equipment including thinner sheaths and improvements in the TAVI BHV delivery systems transfemoral TAVI is now feasible in more than 95% of cases. For those cases unsuitable for transfemoral TAVI, alternative access routes have been developed and adopted, including transapical, transaortic, transcarotid, transaxillary/subclavian, and transcaval approaches, each with different features.

Transapical TAVI was first performed in 2005 and during the early years of TAVI has rapidly emerged as the most frequently used alternative access route for patients with unsuitable iliofemoral arteries. However, due to the increased feasibility of the transfemoral approach, the complications related to the transapical access site as well as the advent of other access routes, transapical access has been substantially declining and it is now rarely used in clinical practice. The transapical approach is associated with increased invasiveness and direct injury to the myocardium, potential respiratory compromise, and an increased recovery time and chest discomfort. Furthermore, the THV choice is restricted only to antegrade delivery systems with an additional risk of apical rupture and pseudoaneurysm formation (69). Observational studies have shown a signal of higher mortality rates in patients treated with transapical compared to transfemoral TAVI (70, 71). Furthermore, propensity score-matched or score-adjusted analyses with a comparison between transapical and transfemoral TAVI after incorporating data derived from studies using an independent event adjudication process suggest a higher short- and long-term mortality, similar 30-day stroke rates, higher rates of major bleeding, and longer length of hospital stay for patients treated with a transapical TAVI approach (72, 73).

Transaxillary or TAVI *via* subclavian access route was first reported in the literature in 2008 (74). The transaxillary approach offers several advantages associated with percutaneous approaches, such as rapid recovery, no myocardial or chest wall injury, no restrictions in patients with prior cardiac surgery, and no interaction with descending or abdominal aorta. In addition, is an attractive approach for obese and extremely obese patients. On the other hand, compared to the femoral artery, the subclavian/axillary arteries are softer and more prone to injury and occlusive dissections. Furthermore, they are not accessible for effective manual compression in case of a bleeding complication and their proximity to the brachial plexus might be linked with a higher risk of upper limb compromise *via* peripheral nerve injury or distal embolism (69). The artery’s minimum diameter should be 6 mm and specific conditions, such as LIMA graft or a pacemaker, should be considered but do not comprise absolute contraindications. A recent meta-analysis with nine observational studies and 2,938 patients showed comparable 30-day mortality between the transfemoral and transaxillary/subclavian access routes with less major vascular complications in the group with the subclavian approach (75). A previous feasibility study recruiting 100 patients undergoing a transaxillary TAVI, in whom access closure was performed with two Perclose ProGlide systems showed that a fully percutaneous transaxillary approach is safe and feasible with successful vessel closure in 94.8% and covered stent treatment in 11% of patients but without any major-access site adverse event being reported. Thirty-day mortality was 6%, life-threatening bleeding was 3%, and no strokes were reported (76).

A more recently introduced access route, the transcaval approach, has emerged as an alternative to the transfemoral and purely percutaneous approach to perform TAVI in patients with prohibitive iliofemoral access routes. The transcaval approach is based on obtaining percutaneous femoral venous access and entering the aorta through the inferior vena cava using an electrified stiff coronary guidewire. Subsequently, microcatheters in a mother and child setup, a stiff guidewire, and eventually, the delivery sheath is inserted. At the end of the procedure, a nitinol occluder device is implanted at the aortic entry site. Multi-sliced CT is crucial for the assessment of the feasibility of this approach as prerequisites involve a sufficiently large calcium-free target zone (≥ 1 cm) of the right abdominal wall and a trajectory free of obstacles (bowel) (69, 77). A recent propensity-weighted analysis of transcaval vs. transaxillary TAVI in contemporary practice showed that patients undergoing transcaval TAVI had lower rates of stroke and similar bleeding compared to those with transaxillary access; however, both approaches were associated with more complications, including worse bleeding, vascular complications, stroke or TIA, intensive care unit and hospital length of stay, and 30-day and 1-year mortality (78). The transcaval strategy has several advantages including the absolute percutaneous nature of the vessel access, no myocardial or chest wall injury and initial access through the distensible femoral vein allows the accommodation of all sheath sizes. In addition, it allows for a standard working position for the operator and thus less exposure to radiation (69). The shortcomings of this approach involve the risk of retroperitoneal bleeding and residual aorto-caval fistula, as well as bowel injury. The development of dedicated devices for aortic entry site closure will probably make this approach more attractive and increase its adoption in clinical practice.

## Transcatheter aortic valve implantation and pertinent adverse events

### Access-site and access-related vascular injury

With the ongoing technical improvements, the access-site and access-related vascular injuries (ASARVI) during TAVI have been substantially reduced over time. However, they remain the most frequent complications, and are associated with worse short- and long-term outcomes (79–82). Most of these complications affect the common femoral and external iliac arteries and among others, they predominantly include access-site bleeding mostly because of closure device failure, vessel dissection, or rupture (82). High body mass index and obese female patients usually have smaller caliber vessels and peripheral vascular disease with calcified atherosclerosis that can result in vascular closure device failure have been all independently correlated with a higher risk of ASARVI (82, 83). The Valve Academic Research Consortium Access-Site and Access-Related Vascular Injury (VARC-2-ASARVI) classification introduced by Sedaghat et al. is a useful tool to easily stratify the severity of vascular injury and proceed to appropriate management (80). The VARC-2-ASARVI is a modified classification model adapted from coronary perforation classification previously introduced by Ellis et al. and stratifies ASARVIs in four major categories: Type I involving blush or minimal dye extravasation; Type II with moderate extravasation (size < 5 mm); Type III with major extravasation (size > 5 mm); and finally, Type IV with acute vessel dissection or occlusion (80).

### Prevention and management of vascular complications

Apart from the refining of arterial access with the introduction of the ultrasound-guided micro-puncture technique significant improvements have also been made regarding the vascular closure techniques and available equipment. Historically, suture-mediated percutaneous vascular closure devices (VCD) have been used for main access closure to avoid surgical cut-down. Among suture based VCD, the Perclose ProGlide (Abbott Vascular) has shown superior results compared to its predecessor Prostar XL (Abbott Vascular), and has become the most used suture-based VCD (84, 85). More recently, a novel large-bore plug-based VCD the MANTA (Teleflex) was introduced aiming to tackle difficult femoral anatomies such as those with higher atherosclerotic burden, where suture-based VCD are more likely to fail. Even though early feasibility trials and retrospective analyses showed promising results, the use of MANTA was associated with higher rates of vascular complications than the double ProGlide technique in two randomized controlled trials (86–89). Initially proposed as a bailout strategy to tackle excessive bleeding, the combined use of a suture-based VCD such as the ProGlide with a plug-based VCD such as the Angioseal (Terumo) has been reported to be safe and feasible (90). The technique involves the insertion of the ProGlide in the beginning before the large sheath insertion followed by the Angioseal insertion at the end of the procedure after the large sheath removal. A recent study has demonstrated a clear superiority of the technique compared to the dual ProGlide technique with significantly reduced main access-related major complications or bleeding ≥ Type 2 according to VARC-3 bleeding classification (3.0 vs. 11.4%) (91).

**FIGURE 6:**
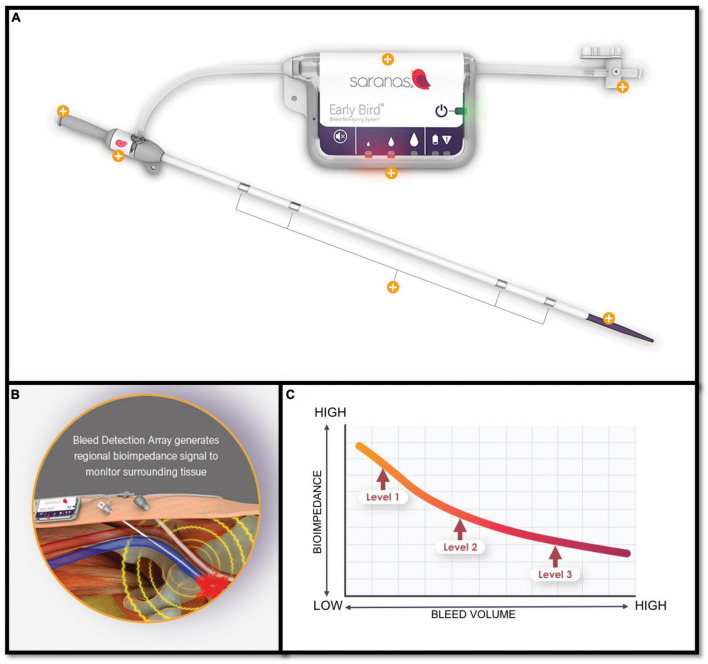
**(A)** The Saranas Early Bird Bleed Monitoring System. **(B)** By monitoring nearby tissue bioimpedance can offer early bleeding detection. **(C)** The lower the bioimpedance the higher the bleeding volume.

Early detection of access-related bleeding complications during TAVI remains challenging as clinical recognition relies on the manifestation of signs and symptoms, such as hematoma, pain, and hypotension and additional imaging confirmation with CT. By the time these bleeding complications become evident with symptoms or are confirmed with imaging, a considerable blood loss has typically already occurred with the subsequent substantial compromise of clinical prognosis. Accordingly, early bleeding detection post-TAVI has become fundamental for patients’ prompt management and survival. A newly introduced device the Saranas Early Bird Bleed Monitoring System (EBMMS) has the capacity to detect bleeding through the continuous measurement of changes in the local bioimpedance (Figure 6). The EBBMS consists of the following parts: (1) A standard vascular access sheath (6 or 8 Fr); (2) four electrodes (two proximal and two distal) that are embedded within the sheath; and (3) a user interface display is integrated on the site of the port of the sheath. A recent study has shown excellent safety profile and accuracy of the device in early detection of bleeding with a high level of agreement with CT scan (Cohen’s Kappa statistic of 0.84, with a sensitivity of 100%, specificity of 75%, a positive predictive value of 98%, and negative predictive value of 100% for bleed detection relative to CT scan findings) (92).

With the occurrence of vascular access, prompt and efficient management is mandatory for achieving adequate bleeding control and a good prognosis. A crossover angiography to assess for aortic/iliofemoral dissection, perforation, or VCD failure is currently the standard practice, and placement of a crossover wire from the contralateral femoral artery allows rapid vascular access with the delivery of the necessary equipment, such as appropriate size balloons to tamponade the area of interest of this deems necessary. Transradial secondary access has recently been demonstrated to be suitable for the management of peripheral vascular complications during TAVR and may reduce the rate of secondary contralateral femoral access complications (93, 94). Limited dissection or perforation can be well managed with prolonged occlusive balloon inflation. Percutaneous deployment of a covered stent or surgical repair is indicated for more extensive flow-limiting dissection or bleeding or in cases with hemodynamic instability or threatened limb circulation. Both options are associated with good outcomes, but the percutaneous option is usually preferred over surgical repair, especially when the injury is above the inguinal ligament as the latter might require laparotomy and TAVI patients are usually old, frail, and have high perioperative risk (80, 95, 96).

### Stroke

#### Incidence of stroke

Despite the device refinements and procedural streamlining, stroke is still a feared and devastating complication of TAVI, which is associated with a 5–10-fold increased risk of mortality (97, 98).

Real-world registries have demonstrated that TAVI procedures have a similar incidence of stroke as SAVR with an in-hospital rate of 1–2% (99). In the STS/ACC TVT registry involving 101,430 patients treated with TAVI between 2011 and 2017, the incidence of stroke was 2.3% (95% CI, 2.2–2.4%), while the transient ischemic attack was reported with a rate of 0.3% (95% CI, 0.3–0.4%) at 30 days. There was no decline in the incidence of stroke over time, indicating that the ongoing technical evolution did not have any positive impact on the prevention of cerebral embolic events. It is worth mentioning that 48.6% of stroke patients experienced a remarkable impairment of social and recreational activities, 34.5% suffered a neurocognitive impairment, and 41.2% required new aids or assistance at the time of event adjudication highlighting the debilitating consequences of stroke after TAVI. Occurrence of stroke was associated with a striking sixfold increased risk of 30-day mortality; HR: 6.1 (95% CI: 5.4–6.8; *P* < 0.001) (100).

In patients undergoing TAVI that belong to the low-risk group, the reported stroke rates appear to be lower. In the PARTNER-3 trial, the 1-year incidence of stroke after transfemoral TAVI was 1.2%, compared to 3.1% after SAVR (HR: 0.38; 95% CI: 0.15–1.00) (26). Interestingly, at 2-year follow-up, the investigators reported a convergence of stroke rates without a significant difference between TAVR and SAVR cohorts (2.4 vs. 3.6%, *p* = 0.28). This is more likely related to a plausible higher rate of THV thrombosis after TAVI (28). In the low-risk group treated with either the self-expandable platform CoreValve/Evolute or SAVR, the incidence of stroke was similar between TAVI and SAVR at 1-year follow-up namely 4.1 vs. 4.3% (27).

With regards to the rates of stroke specifically related to the device platform either the self-expandable or the balloon-expandable devices, the results are rather conflicting. A previous large propensity-matched population of 8,192 patients from the CENTER collaboration found a lower stroke incidence at 30-days in the balloon-expandable cohort for SAPIEN XT/3 vs. CoreValve/Evolute: 1.9 vs. 2.6% (*p* = 0.03) (101). In contrast, in the more recent SOLVE TAVI trial, a direct randomized comparison of 447 patients treated with transfemoral TAVI, with either Evolute-R or Sapien-3, a numerically lower stroke rate of 0.5% for self-expandable THVs compared with 4.7% for balloon-expandable was observed without reaching statistical significance in the superiority testing (35).

#### Intra-procedural measures to prevent stroke

The use of cerebral embolic protection devices has intuitively emerged as a new tool that could potentially reduce cerebral embolic events during and after a TAVI procedure. However, the currently available data have not demonstrated any robust and clear benefit from regular use of this specific equipment. A recent meta-analysis (102) failed to demonstrate a reduction in the incidence of stroke or the mean number of silent brain infarcts per patient. In addition, these devices appear to be used infrequently as this was shown in the German registry of 41,654 TAVIs, whereas cerebral embolic protection devices were used in 3.8% of cases. Moreover, the use of these devices did not reduce the risk of stroke or the risk of developing delirium as a sign of acute brain failure (103). In propensity-matched score analysis of patients undergoing TAVI, the use of cerebral protection devices demonstrated a significantly higher rate of stroke-free survival compared to unprotected patients (104). In contrast, the SENTINEL pivotal trial and the CLEAN TAVI trial failed to demonstrate a marked reduction in rates of clinically significant stroke associated with TAVI (105, 106). Moreover, in the intention to treat analysis of the CLEAN TAVI trial the incidence of new neurological symptoms indicating an acute stroke was 10% in both the SENTINEL protection and the unprotected group of patients (105). In the largest so far RCT, including 3,000 patients, the PROTECTED TAVR trial, the use of the SENTINEL cerebral protection device was not associated with a significant reduction of periprocedural stroke during TAVI (107). Another ongoing RCT the BHF PROTECT TAVI trial (ISRCTN16665769) involving 7,730 patients undergoing TAVI with a direct comparison between patients with and without an intraprocedural SENTINEL cerebral protection device deployment will shed more light regarding the protective effect of the systematic use of cerebral protection devices in preventing the incidence of clinically evident stroke after TAVI.

To conclude, although cerebral protection devices have been proven efficient in drastically reducing the new ischemic brain lesions post-TAVI (105, 108), clear, robust, and groundbreaking results in preventing clinically evident strokes are still missing.

#### New conduction abnormalities and permanent pacemaker implantation after transcatheter aortic valve implantation

##### Pathophysiology

**FIGURE 7:**
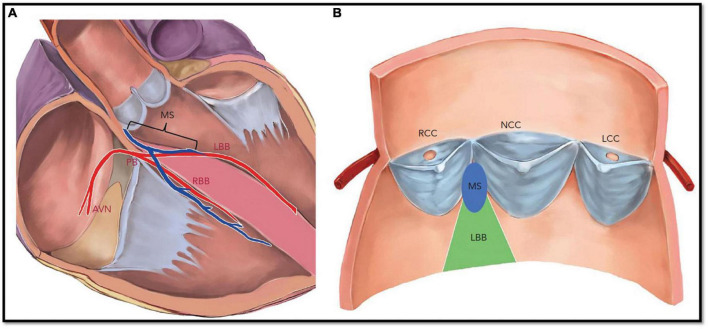
Native cardiac conduction system and its anatomical relations with aortic valve cusps and membranous septum. **(A)** The penetrating bundle of His emerges at the surface of the left ventricular outflow tract beneath the membrane septum (MS). The length of the MS is equal to the distance between the aortic annulus and the bundle of His. **(B)** The left bundle branch emerges beneath the MS and is positioned between the right coronary cusp and non-coronary cusp. AVN, atrioventricular node; LBB, left bundle branch; LCC, left coronary cusp; PB, penetrating bundle; MS, membrane septum; NCC, non-coronary cusp; RBB, right bundle. Reproduced from Lin et al. (159).

The aortic valve has a close spatial proximity to the intrinsic conduction system of the heart. In particular, the atrioventricular node (AVN) is near the subaortic region where the His bundle is running on the lower edge of the membranous septum in the left ventricular outflow tract (LVOT). TAVI prostheses are inserted in an intra-annular position and in contrast to surgical valves, which entail exerting pressure against the aortic annulus to maintain the stent frame in the desirable position (Figure 7). Excessive THV over-sizing can inadvertently compress the cardiac conduction system, which can subsequently cause transient or permanent mechanical damage to the surrounding tissue involving edema, hematoma, or necrosis of the conduction system. Almost half of these disorders may improve over time and will not require PPM due to the resolution of the associated trauma (109).

##### Incidence and risk factors associated with new conduction abnormalities after transcatheter aortic valve implantation

The most encountered conduction abnormalities after TAVI are a high degree or complete atrioventricular block requiring PPM implantation or new onset left bundle branch block (LBBB). Over the years, the incidence of PPM implantation and new conduction abnormalities have markedly decreased in line with the adoption of new-generation THVs and the implementation of novel implantation techniques. Historically, self-expanding Evolute R and PRO have demonstrated a higher percentage of conduction abnormalities compared to the balloon-expandable devices Sapien 3 and Sapien 3 ULTRA (12–20% PPM, 18–28% LBBB, vs. 4.4–6.5% PPM, and 13–24% LBBB) primarily due to different depths of implantation and mechanisms of expansion between the two types of valves (26, 27, 110, 111). Other more recent devices such as the Acurate-neo valve and the Portico with FlexNav system have shown acceptable PPM implantation rates of 10 and 14.6%, respectively. In the most recent PARTNER III Trial, 6.6% of the overall low-risk TAVI population required treatment with PPM implantation, which was found to be comparable with the corresponding rate of PPM implantation in the SAVR group. However, more patients in the TAVI group developed new LBBB than SAVR patients (22 vs. 8%) (26). In contrast, the Evolut Low-Risk TAVI Trial with self-expanding THVs showed that TAVI patients underwent postoperative permanent pacemaker (PPM) implantation much more frequently compared to SAVR individuals (17.4 vs. 6.1%, respectively) (27). It is felt that the unremittingly increased radial force applied on the wall of the left ventricular outflow tract associated with the self-expanding platforms might explain the higher rates of PPM implantation with the self-expanding THVs, compared to the balloon-expandable THVs (112).

Baseline electrocardiographic findings, anatomical features such as shorter membranous septum length (MSL), LVOT eccentricity, and severe annular calcification have been identified as potential risk factors for developing significant conduction abnormalities and subsequent need for PPM after TAVI (113–115). The presence of baseline right bundle branch block (RBBB) represents the most observed electrocardiographic predictor of PPI with an increased risk from 3 up to 47 times (115). With regard to other procedural factors, implant depth has been identified as the strongest and most consistent predictor among procedural factors. The adoption of a new THV implantation technique for the self-expanding system of EVOLUTE, known as the cusp-overlap view implantation technique (coplanar projection by overlapping the right and left coronary cusps) has enabled a higher implantation depth and compared to the conventional 3-cusp view implantation, has shown remarkable results with significant decrease in the 30-day new-onset LBBB (12.9 vs. 5.8%; *p* = 0.005) and PPM implantation rate (17.8 vs. 6.4%; *p* = 0.004), without any differences in MACE rate (116, 117). A similar approach with a high deployment technique for the balloon-expandable THV Sapien 3 has achieved a substantial decrease in a 30-day PPM implantation rate and the incidence of new-onset conduction abnormalities (118).

Two recent studies have evaluated the role of post-TAVI long-term monitoring with an implantable loop recorder (ILR) to appropriately recognize late clinically significant, high-degree conduction abnormalities or other arrhythmias, such as atrial fibrillation or ventricular tachycardia. In the first study, 98 patients undergoing a TAVI procedure (42% self-expanding THVs, 53% balloon-expandable valves) received an ILR (31% a median 20 days before TAVI and 69% a median 1 day after TAVI) with a follow-up at 1-year. Of the study participants, 7 and 10% had pre-existing right (RRBB) and LBBBs, respectively. LBBB increased to 39% post-TAVI and decreased to 22% after 1 year. A PPM was implanted in 15 out of 98 (15%), of which nine (60%) received the PPM before discharge. Of the six patients receiving the PPM after hospital discharge, three patients (3% of the overall cohort) developed complete heart block and this occurred within maximum 14 days after TAVI. The other three patients received a PPM because of sick sinus syndrome. This study highlights that many conduction abnormalities related to TAVI occur within the first 2 days post TAVI usually before patients’ discharge, while after 2 weeks post-TAVI high degree atrioventricular block related to TAVI is extremely unlikely to occur (119).

In the MARE study, 103 consecutive patients undergoing TAVI (50% balloon expandable BHV) and new persistent LBBB post-TAVI received an ILR. At 1-year follow up significant bradyarrhythmia, including severe bradycardia or high-degree AV block occurred in 20% of patients. In 10% of the patient, treatment with PPM implantation was required. Of those, 50% received the PPM due to high-degree AV block within the first 18 days after TAVI, while the rest 50% within 7 months post-TAVI highlighting that new persistent LBBB post-TAVI might require closer short and long-term monitoring due to a higher likelihood for advanced high-degree AV block.

##### Conduction disturbances after transcatheter aortic valve implantation and associated prognosis

The data regarding the impact of new conduction abnormalities and PPM implantation on prognosis after TAVI remain controversial. A recent study in intermediate-risk patients undergoing TAVI showed that new-onset LBBB was associated with a significant increase in all-cause and cardiovascular mortality, rehospitalization, PPM implantation, and decreased LV function at 2 years (120). Additional studies have shown that new-onset LBBB after TAVI is an independent predictor of all-cause mortality at more than 2 years of follow-up (121, 122). A recent meta-analysis of 12 studies reported an increased risk of all death at 1 year of follow-up in patients with a new persistent LBBB post-TAVI [RR: 1.32; 95% CI (1.17–1.49); *P* < 0.001]. In addition, new LBBB was associated with a higher risk of cardiac death [RR: 1.46; 95% CI (1.20–1.78); *P* < 0.001], heart failure requiring hospitalization [RR: 1.35; 95% CI (1.05–1.72); *P* = 0.02), and 1-year PPM [RR: 1.89; 95% CI (1.58–2.27); *P* < 0.001] at 1-year follow up (123). In contrast, two other studies and a meta-analysis did not show any relation of new-onset LBBB with 1-year all-cause mortality (124–126). Rodes-Cabau et al. recently proposed an algorithmic approach to the management of new LBBB post-TAVI. Patients with persistent LBBB at day 2 with QRS ≤ 150 ms and PR ≤ 240 ms could be safely discharged and continuous ECG monitoring (2–4 weeks) could be considered. Patients with QRS > 150 ms and PR > 240 ms are at increased risk of delayed high-degree AV Block and continuous ECG monitoring or electrophysiology studies might be considered to guide a decision for prophylactic PPM insertion. If further QRS or PR interval prolongation of ≥ 20 ms within 24 h was observed, then evaluation with an electrophysiology study followed by continuous ECG monitoring or direct PPM insertion might be considered (127).

Similarly, the clinical impact of PPM insertion after TAVI remains also controversial. Right ventricular pacing has been associated with inter- and intraventricular desynchrony and can intuitively result in detrimental effects on cardiac structure and overall myocardial contractility and function. RV pacing has been shown to cause left ventricular remodeling heart failure and death (128, 129). Results from the TVT registry have consistently shown that PPM implantation after TAVI has been associated with increased mortality (112, 120, 130). Furthermore, Costa et al. have shown that post-TAVI patients with PPM dependence showed higher overall mortality compared to the non-dependent patients (131). In contrast, a meta-analysis of 7,032 patients showed that periprocedural PPM after TAVI was not associated with an increased risk for all-cause mortality at 1-year (126). In another multicenter trial with 1,629 patients undergoing TAVI 19.8% required a PPM insertion. Even though PPM insertion is associated with a higher risk for heart failure hospitalization, there were no differences in all-cause and cardiovascular mortality between those with and without a PPM (132). These contradictory results can be attributed on one hand to the detrimental effects of pacing dependence on overall cardiac structure and function with patients that are not pacing dependent being less likely prone to develop adverse outcomes and on the other hand the protective effect of pacemakers against sudden cardiac death.

#### Coronary access and occlusion after transcatheter aortic valve implantation

##### Coronary access after transcatheter aortic valve implantation

The high prevalence of concomitant coronary artery disease in patients with AS, almost 50% (133), as well as further expansion of TAVI indications in low-risk and younger patients are critical factors that should be taken into account in all TAVI candidates. In this regard, it is critical to aim for seamless and uncomplicated coronary access after TAVR allowing for future diagnostic coronary angiograms, as well as percutaneous coronary intervention. In a recent study evaluating the impact of acute coronary syndromes (ACS) in 779 patients following TAVI, approximately 10% of the overall cohort of patients were readmitted with ACS after a median follow-up of 2 years. The presentation involved type 2 MI in 36% of patients, unstable angina in 35%, NSTEMI in 28%, and STEMI in 1% with associated mortality at 2 years post-ACS of 37% (134). The difficulty of coronary re-access post-TAVI is correlated with the implanted bioprosthesis design: it is considerably easier with the short-stent frame and sub-coronary position balloon-expandable platforms, and it is more difficult with the supra-annular THVs with the tall stent frames and small struts. However, previous reports have shown unsuccessful coronary cannulation in 9–13% of patients treated with SAPIEN THV as well, which is not negligible, especially in low-risk young patients that might require coronary intervention in the future (135). In the REVIVAL (Revascularization After TAVI) study, PCI was successfully executed after TAVI in 96.6% of patients, without any significant differences between THV designs (136). In the RE-ACCESS (Reobtain Coronary Ostia Cannulation Beyond TAVI) study among 300 patients that were enrolled, unsuccessful coronary cannulation following TAVI was seen in 7.7% of cases. The use of Evolut R/PRO THVs, the THV implant depth, and the oversizing of the THV in relation to the sinus of Valsalva diameter were independent factors associated with unsuccessful cannulation of the coronaries (137). On the other hand, data from the RESOLVE registry with a real-world cohort of patients have shown unfavorable coronary access in up to 35% of patients after TAVI, as assessed with post-implantation CT angiograms in 66 patients. The authors concluded that THVs with a low skirt and commissural height pattern and large open cells that are specially designed to achieve commissural alignment with the native aortic valve may facilitate future coronary access (138). In addition, a simulation study predicted that sinus of Valsalva sequestration and resultant coronary obstruction will occur in up to 23% of patients treated with Evolut-Pro during future TAVI in TAVI procedures (139). That was the case for SAPIEN prostheses as well, where the most challenging anatomies for post-TAVI coronary cannulation including THV stent frame above the coronary ostia and commissural suture position in front of a coronary ostium were observed in 9–13% of patients (135). The alignment of Transcatheter Aortic-Valve Neo-Commissures (ALIGN TAVR) studies first evaluated the impact of THV deployment orientation on neo-commissural overlap with coronary arteries. In this pilot imaging study 828 TAVR implants (SAPIEN 3 = 483, Evolut = 245, ACURATE -neo = 100) were analyzed using pre-procedural multidetector row CT and coplanar fluoroscopy co-registration. While different crimping orientations of the SAPIEN 3 THV did not result in consistent commissural alignment, specific flush port positioning significantly influenced the rate of neo-commissural alignment with Evolut THV. Evolut flush port positioned at 3 o’clock improved “hat” marker orientation to the outer curve or center front at the annulus, thus reducing the rate of coronary artery overlap from 60 to 36%; *p* < 0.05 compared to that marker positioned toward the inner curve or center back. ACURATE-neo commissure positioning at the center back/inner curve significantly improved commissural alignment compared to the center front or outer curve (140, 141).

In line with the application of TAVI in younger patients with a potential need for coronary intervention, the implanting team should focus on three major technical aspects:

1. A THV with a sub-coronary frame position is generally preferable.
2. Commissural alignment is mandatory when a supra-annular valve design is used especially in narrow roots.
3. THVs with large open cells are beneficial for stents that cover the coronary ostia.

##### Acute coronary occlusion after transcatheter aortic valve implantation

Since the introduction of dedicated CT TAVI as gold-standard in the routine pre-procedural planning of TAVI, acute coronary occlusion is an uncommon complication following TAVI, with a reported incidence of < 1% (142, 143). The left main is mostly involved, encountered in approximately 87% of cases of coronary obstruction (142, 143). Well-recognized risk factors include the short distance between the annulus and coronary ostia < 10 mm and a narrowed aortic root < 28 mm at the level of sinuses of Valsalva (142, 143). Both these factors increase the risk of displacement of the native aortic valve leaflets over the coronary ostia with subsequent acute or late coronary occlusion. This risk becomes even higher during ViV TAVIs with a risk of acute coronary occlusion of 2.3% with a rate of 30-day mortality up to 50% (144). Different strategies have been developed to prevent this dreadful complication. In selected patients, the preventive strategy of placing an under-deployed stent in coronary artery ostia (Chimney stenting) has been reported as a simple and effective technique to prevent acute coronary occlusion after TAVI in patients at high-risk (145). Although the data regarding the efficacy of chimney stenting are reassuring, there are concerns regarding the risk of late stent failure (3.5% at 1 year). The Bioprosthetic or Native Aortic Scallop Intentional Laceration to Prevent Iatrogenic Coronary Artery Obstruction (BASILICA) has emerged as a novel technique to prevent post-TAVI acute or late coronary artery occlusion. Based on intentional laceration of preexisting native or bioprosthetic aortic valve leaflet in front of the threatened coronary artery, BASILICA appears achievable with a procedural success rate of 87% and relatively safe with a 30-day mortality of 2.8% (146). However, the extensive toolbox that is required to perform the procedure and the complex and high-risk nature of the procedure itself dictates the need for further refinement of this technique to facilitate its wider clinical adoption.

#### Paravalvular leak

Paravalvular leak is generally a result of inappropriate valve sealing and incomplete apposition between the THV and the aortic annulus and is contingent on specific THV designs. Since the introduction of TAVI, the rate of PVL used to be frequent. Moreover, moderate, or severe PVL has been recognized as a strong independent predictor of mortality (147, 148). Risk factors for PVL include severe native aortic valve calcification, leaflet asymmetry, prosthesis malapposition or undersized, and self-expanding valves. Self-expanding valves exert less radial force compared to balloon-expandable valves, whereas excessive annular calcification has a more prominent effect on the final configuration of self-expanding valves, with frequent under-expansion and eccentric post-deployment shape of the latter (149). The evolution in THV and the subsequent improved operator experience has led to a remarkable decline in rates of PVL over time. A progressive reduction for moderate and severe PVL has been observed throughout RCTs up to 0.8% in the PARTNER 3 trial and 3.5% in the Evolut low-risk trial at 30 days (26, 27). On the contrary, no discernable change has been demonstrated regarding mild PVL, whose prognostic impact remains undefined. Recent data have shown a reduction for mild PVL with the latest generation balloon-expandable SAPIEN 3 Ultra compared to SAPIEN 3 THV (none-trace PVL 90.9 vs. 85.7%; *p* < 0.01 and mild PVL 8.9 vs. 13.9%; *p* < 0.01). Similarly, newer generation Evolut PRO had lower rates of mild PVL compared to Evolut R THV (none-trace PVL: 70.3 vs. 63.2% and mild PVL 27.8 vs. 34.8%; *p* = 0.007). In the SCOPE I trial ACURATE-neo showed higher rates of moderate-severe PVL compared to SAPIEN 3 THV (9.4 vs. 2.8%; *p* < 0.001). These findings were further confirmed in the SCOPE 2 trial where ACURATE-neo was compared to EVOLUT R (10 vs. 3%; *p* = 0.002). Balloon post dilatation or TAVI in TAVI has been described as a potential option to treat moderate-severe PVL. Other options include the percutaneous closure with plugs, with good overall results in terms of safety and efficacy (150).

#### Subclinical transcatheter heart valve thrombosis

Since TAVI introduction and subsequent wider adoption for the treatment of symptomatic severe AS several concerns were raised regarding the thrombogenicity of the THVs and therefore the systematic treatment with antiplatelet therapy was endorsed. In 2015, the ongoing success of TAVI was intercepted by the worrisome report of the phenomenon of subclinical leaflet thrombosis (151). A systematic protocol based on 4D high-resolution CT imaging is currently available for the evaluation and classification of the different patterns of subclinical THV leaflet thrombosis. The key CT features that were noted involved the hypoattenuating leaflet thickening (HALT) associated with reduced leaflet motion (RELM) leading to hypoattenuation affecting motion (HAM). The stratification of severity of RELM was further allocated to moderate (50–69%), severe (70–99%), and immobile (152). So far, no significant clinical implications of these CT findings have been shown, while the incidence of subclinical leaflet thrombosis appears to be comparable between TAVI and SAVR. In the pre-specified analysis of the Evolut Low-Risk CT sub-study among 179 patients undergoing TAVI, not oral anticoagulation therapy, HALT and RLM occurred frequently (HALT, 17.3% at 30 days and 30.9% at 1 year; RLM, 14.6% at 30 days, and 31% at 1 year) without any significant difference with SAVR patients. The detection of subclinical leaflet thrombosis was not associated with THV gradient or any clinical events (153). In a similar CT sub-study from the PARTNER 3 trial subclinical bioprosthetic valve leaflet thrombosis occurred more frequently in the TAVI group compared to the SAVR group at 1 month (TAVI: 13% vs. SAVR: 5%, *p* = 0.03), with a convergence of the incidence of subclinical leaflet thrombosis between the two groups at 1 year (TAVI: 28% vs. SAVR: 20%; *p* = 0.19) with no significant difference in the transvalvular gradient between the two groups. In addition, no association of HALT with death, stroke, or MI was observed. However, patients with more excessive HALT demonstrated an increase in thromboembolic events, while 1-year persistent HALT was associated with a higher mean transvalvular gradient (∼ 5 mmHg) (154). The lower incidence of subclinical valve leaflet thrombosis in the SAVR group might at least in part be explained by the potentially higher proportion of these patients on oral anticoagulation therapy due to other clinical conditions, such as atrial fibrillation. In the GALILEO (Global Study Comparing a Rivaroxaban-Based Antithrombotic Strategy to an Antiplatelet Strategy After Transcatheter Aortic Valve Replacement to Optimize Clinical Outcomes) trial, the group of patients on rivaroxaban demonstrated significantly less HALT compared to those on antiplatelet only therapy (155). However, patients on anticoagulation showed higher mortality rates, a warning sign indicating that patients with severe AS represent a heterogenous group of patients with multiple underlying comorbidities and a complex interaction between high bleeding and ischemic risk that makes the choice of the appropriate antithrombotic treatment even more complex. In the ENVISAGE study, a multicenter RCT with 1,426 patients undergoing TAVI with atrial fibrillation and a primary indication for anticoagulation the patients were randomized to either therapeutic treatment with edoxaban or warfarin. Patients on edoxaban had higher rates of major bleeding compared to patients on warfarin (hazard ratio: 1.4; 95% CI: 1.03–1.91; *p* = 0.93 for non-inferiority) without any significant difference regarding the rates from any cause or stroke (156). In the most recent ATLANTIS trial, 1,500 patients undergoing TAVI were randomized to either oral anticoagulation with apixaban 5 mg od or standard-of-care therapy, which included either a vitamin-K antagonist if there was a primary indication for anticoagulation or antiplatelet therapy. There was no difference between the groups regarding the primary composite endpoint of death, myocardial infarction, stroke or transient ischemic attack, systemic embolism, intracardiac or bioprosthesis thrombosis, deep vein thrombosis or pulmonary embolism, and life-threatening, disabling, or major bleeding over 1-year follow up (18.4 vs. 20.1%) without any evidence of interaction between any treatment (apixaban, vitamin-K antagonist or antiplatelet therapy—*p* interaction = 0.57). Moreover, the primary safety endpoint of major, disabling, or major bleeding over 1-year follow-up was not different between the groups. Interestingly, in the study stratum of 1,049 patients where apixaban was compared to antiplatelet therapy only, therapeutic apixaban was associated with significantly less obstructive valve thrombosis (HR: 0.19, 95%CI: 0.08–0.46), while a signal of higher non-cardiovascular mortality that was observed with apixaban (157). Finally, in the ADAPT-TAVR study with 229 patients undergoing TAVI and without any indications for anticoagulation, edoxaban resulted in numerically twofold lower subclinical THV leaflet thrombosis at 6 months (9.8 vs. 18.4%). However, the rates of death, stroke, transient ischemic attack, blood clotting in the brain and neurocognitive dysfunction were not different between the groups (158). Until further long-term follow-up results become available to further elucidate whether the reduction of THV thrombosis with oral anticoagulation will eventually be translated to overt clinical benefits, the primary antithrombotic therapy unless there is another indication for oral anticoagulation should include a single antiplatelet regimen with aspirin or clopidogrel.

## Conclusion and future directions

Twenty years after the first breakthrough procedure, TAVI underwent a transformative evolution and currently can be unanimously considered the most striking development in the field of interventional cardiology for the twenty-first century. A lifesaving procedure that was initially developed to treat inoperable and terminal patients with critical AS has now established itself as the treatment of choice for most patients with severe symptomatic AS. As the number of patients that will have an indication for TAVI is likely to further grow with broader expansion and timing for intervention in currently ambiguous scenarios, including moderate AS with heart failure (TAVR UNLOAD trial, NCT02661451), asymptomatic severe AS (EARLY TAVR study, NCT03042104), bicuspid AS and native aortic regurgitation further refinements in the technology behind the THVs and implanting techniques will be necessary to completely eliminate adverse events such as the need for PPM implantation and further improve other aspects such as THV durability and post-TAVI coronary access (Figure 4).

## Author contributions

AK and TP made the initial manuscript conception and structure and the initial manuscript draft and further edited and approved the final version of the manuscript. SR, CA, HH, OC, RR, and BP edited and approved the final version of the manuscript. All authors contributed to the article and approved the submitted version.
